# Products of Vitamin D3 or 7-Dehydrocholesterol Metabolism by Cytochrome P450scc Show Anti-Leukemia Effects, Having Low or Absent Calcemic Activity

**DOI:** 10.1371/journal.pone.0009907

**Published:** 2010-03-26

**Authors:** Andrzej T. Slominski, Zorica Janjetovic, Brian E. Fuller, Michal A. Zmijewski, Robert C. Tuckey, Minh N. Nguyen, Trevor Sweatman, Wei Li, Jordan Zjawiony, Duane Miller, Tai C. Chen, Gerard Lozanski, Michael F. Holick

**Affiliations:** 1 Department of Pathology and Laboratory Medicine, University of Tennessee Health Science Center, Memphis, Tennessee, United States of America; 2 Department of Molecular Enzymology, Intercollegiate Faculty of Biotechnology University of Gdansk and Medical University of Gdansk, Gdansk, Poland; 3 School of Biomedical, Biomolecular and Chemical Sciences, University of Western Australia, Crawley, Western Australia, Australia; 4 Department of Pharmacology and the Center for Cancer Research, University of Tennessee Health Science Center, Memphis, Tennessee, United States of America; 5 Department of Pharmaceutical Sciences, College of Pharmacy, University of Tennessee Health Science Center, Memphis, Tennessee, United States of America; 6 Department of Pharmacognosy and Research Institute of Pharmaceutical Sciences, School of Pharmacy, University of Mississippi, University, Mississippi, United States of America; 7 Division of Endocrinology, Department of Medicine, Boston University School of Medicine, Boston, Massachusetts, United States of America; 8 Department of Pathology and Laboratory Medicine, State University of Ohio, Columbus, Ohio, United States of America; Ludwig-Maximilians-University, Germany

## Abstract

**Background:**

Cytochrome P450scc metabolizes vitamin D3 to 20-hydroxyvitamin D3 (20(OH)D3) and 20,23(OH)_2_D3, as well as 1-hydroxyvitamin D3 to 1α,20-dihydroxyvitamin D3 (1,20(OH)_2_D3). It also cleaves the side chain of 7-dehydrocholesterol producing 7-dehydropregnenolone (7DHP), which can be transformed to 20(OH)7DHP. UVB induces transformation of the steroidal 5,7-dienes to pregnacalciferol (pD) and a lumisterol-like compounds (pL).

**Methods and Findings:**

To define the biological significance of these P450scc-initiated pathways, we tested the effects of their 5,7-diene precursors and secosteroidal products on leukemia cell differentiation and proliferation in comparison to 1α,25-dihydroxyvitamin D3 (1,25(OH)_2_D3). These secosteroids inhibited proliferation and induced erythroid differentiation of K562 human chronic myeloid and MEL mouse leukemia cells with 20(OH)D3 and 20,23(OH)_2_D3 being either equipotent or slightly less potent than 1,25(OH)_2_D3, while 1,20(OH)_2_D3, pD and pL compounds were slightly or moderately less potent. The compounds also inhibited proliferation and induced monocytic differentiation of HL-60 promyelocytic and U937 promonocytic human leukemia cells. Among them 1,25(OH)_2_D3 was the most potent, 20(OH)D3, 20,23(OH)_2_D3 and 1,20(OH)_2_D3 were less active, and pD and pL compounds were the least potent. Since it had been previously proven that secosteroids without the side chain (pD) have no effect on systemic calcium levels we performed additional testing in rats and found that 20(OH)D3 had no calcemic activity at concentration as high as 1 µg/kg, whereas, 1,20(OH)_2_D3 was slightly to moderately calcemic and 1,25(OH)_2_D3 had strong calcemic activity.

**Conclusions:**

We identified novel secosteroids that are excellent candidates for anti-leukemia therapy with 20(OH)D3 deserving special attention because of its relatively high potency and lack of calcemic activity.

## Introduction

In vivo, vitamin D3 (D3) is generated in the epidermis through photochemical transformation of 7-dehydrocholesterol (7DHC) after absorption of ultraviolet radiation B (UVB; wavelength 280–320 nm) energy [1]–[4]. The active form of vitamin D3, 1,25(OH)_2_D3, is generated systemically through sequential 25- and 1α– hydroxylation in the liver and kidney, respectively [4], [5]. It is also produced locally in different organs including skin [5], [6]. 1,25(OH)_2_D3, in addition to being a major regulator of body calcium homeostasis, also inhibits cell proliferation, stimulates differentiation and/or apoptosis, has tumorostatic and anticarcinogenic properties, and protects DNA against oxidative damage [4], [7]–[10]. Importantly, 1,25(OH)_2_D3 and its derivatives also display potent anti-leukemic activities [11]–[17]. Different leukemia lines serve as excellent models for testing vitamin D analogs. Unfortunately, the toxicity (hypercalcemia) of high levels of vitamin D largely prevents the use of pharmacological doses of 1,25(OH)_2_D3 for either the prevention or treatment of cancer. Therefore, there is a continuing search to find vitamin D analogues (for example, Gemini analogs) that retain antiproliferative activity but that are non-calcemic, acting as partial receptor agonists for the VDR [18]–[20]. In addition, more than 30 years ago Holick et al showed that shortening the side chain of vitamin D or eliminating it (producing 20-hydroxypregnacalciferol (20(OH)pD), attenuates or eliminates the calcemic effect [21].

Cytochrome P450scc hydroxylates vitamin D3 in a sequential manner, predominantly to 20-hydroxyvitamin D3 (20(OH)D3). Although further hydroxylation producing 20,23(OH)_2_D3 and other metabolites also occurs, these hydroxy-derivatives represent a minority of reaction products [22]–[25]. 20(OH)D3 can also be produced by P450scc in purified adrenal mitochondria [22] and acts as a potent inducer of keratinocytes differentiation [26] and inhibitor of NF-κΒ activity [27]. P450scc also hydroxylates 1-hydroxyvitamin D3 to 1,20-dihydroxyvitamin D3 (1,20(OH)_2_D3) a product that shows antiproliferative activity towards human keratinocytes [28]. Finally, P450scc in vitro or ex-vivo in adrenal glands, cleaves the side chain of 7-dehydrocholesterol (7DHC) producing 7-dehydropregnenolone (7DHP), which can undergo further oxidations by steroidogenic enzymes [29], [30]. 7DHP and it hydroxyderivatives, can undergo UVB-induced transformation to corresponding pregnacalciferol (pD) and lumisterol-like (pL) compounds [30]–[32]. These secosteroids with only a two-carbon side chain (pD and 20(OH)pD) have been shown to lack calcemic activity in vivo [21].

Thus, having a large panel of novel secosteroidal compounds that can be biologically active but do not have a side chain or have a side chain modified at C20 (similar to the Gemini type analogues), we decided to test whether any of these novel compounds could act as relatively non-toxic anti-leukemic agents.

## Results

### 1. Structures and origin of novel secosteroids (see Figure S1)

Vitamin D3, produced through photolytic transformation of 7-DHC, is hydroxylated to 1,25(OH)_2_D3 (calcitriol) by the sequential actions of 25- and 1α-hydroxylases [8]. Vitamin D3 is also hydroxylated by P450scc to 20(OH)D3 and 20,23(OH)_2_D3 [22], [23], while 1,20(OH)_2_D3 is generated through hydroxylation of 1(OH)D3 by P450scc [28] or by the action of 1α-hydroxylase on 20(OH)D3 [33]. 20(OH)D3 can also derive from UVB induced photolytic transformation of 20(OH)7DHC that is produced by hydroxylation of 7DHC by P450scc [29]. 20(OH)7DHC is further transformed to 7DHP by P450scc [29], [30] then after absorption of UVB photons forms pD or pL depending on the UV energy [32]. 7DHP is potentially reduced by 20-hydroxysteroid dehydrogenase to 20(OH)7DHP [30] which undergoes UVB induced transformation to 20(OH)pD [31].

### 2. Novel secosteroids have low calcemic effect

**Figure 1:**
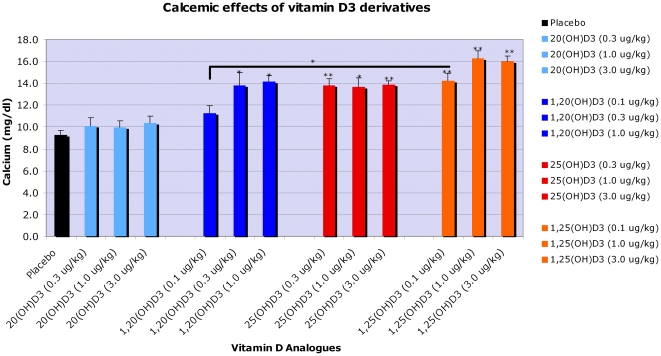
20(OH)D3 and 1,20(OH)_2_D3 have less calcemic effect in comparison to 1,25(OH)_2_D3. In vivo testing of calcemic effects of 20(OH)D3 and 1,20(OH)_2_D3 in comparison to 1,25(OH)_2_D3.

One of us (MFH) has documented that elimination of all but two carbons of the side chain in vitamin D3, leading to production of 20(OH)pD and pD, eliminates the calcemic effect of the secosteroid [21]. Since P450scc hydroxylates the side chain of D3 and 1(OH)D3 at C20, we tested the calcemic effects of the resulting products in rats at a doses 0.1 µg/kg–3 µg/kg of body weight (Fig. 1). 20(OH)D3 at dose as high as 3.0 µg/kg had no calcemic activity (calcium  =  10.4±1.5 vs 9.3±1.3 mg/dL for control), whereas 1,25(OH)_2_D3 at the same dose had the expected strong calcemic effect raising calcium to 16.0±1.2 mg/dL. Interestingly, addition of a 1α-hydroxyl group to 20(OH)D3 promoted calcemic activity raising calcium to 13.9±0.8 mg/dL. Thus for 20(OH)D3, the lack of 1α-hydroxylation underlies its inability to influence serum calcium. 1,20(OH)_2_D3 displays a calcemic effect, however less than that of 1,25(OH)_2_D3. This is consistent with a fundamental role of the hydroxyl group at the 1 alpha-position of vitamin D for the regulation of serum calcium levels [8], [21].

### 3. Novel secosteroidal products show anti-leukemic activity

Anti-leukemic effects of 20(OH)D3, 20,23(OH)_2_D3, 1,20(OH)_2_D3, pD, 20OHpD and pL in comparison to 1,25(OH)_2_D3 and to precursor steroidal 5,7-dienes (for structures see Figure S1), were tested in K562 human chronic myeloid and MEL mouse erytholeukemia and human HL-60 promyelocytic and U937 promonocytic leukemia cells. The choice of 10^−7^ M as the concentration of drugs to use in the majority of experiments was based on initial experiments (not shown) as well as on already published data [26], [27], [31], [34], demonstrating that this concentration is optimal for comparative studies.

#### Cell proliferation

**Figure 2:**
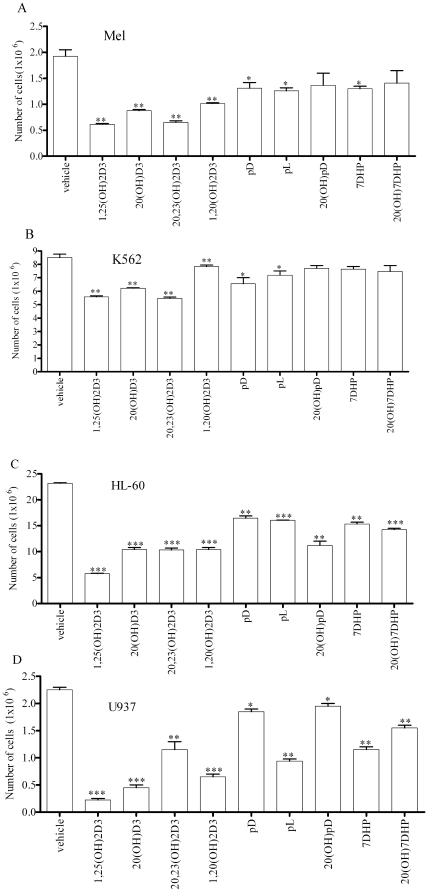
Novel secosteroids and steroidal 5.7-diene compounds have anti-proliferative effects on leukemia cells. The anti-proliferative effects of novel secosteroidal and steroidal 5,7-diene compounds on Mel mouse erythroleukemia (**A**), human K562 myeloid (**B**), HL-60 human promyelocytic (**C**), U937 promonocytic (**D**) leukemias. After treatment for 7 days with different compounds (10^−7^ M), cells were stained with trypan blue and the number of viable cells counted. Statistically significant effects are marked as follow *p<0.05; **p<0.01; ***p<0.001.

The secosteroids inhibited cell proliferation, however, with different potencies in different cell lines. Specifically, in mouse (Mel) erythroleukemia the strongest inhibitory effects were seen for both 1,25(OH)_2_D3 and 20,23(OH)_2_D3 with slightly lower (p<0.05) but strong effects for 20(OH)D3 (p<0.01) (Fig. 2A) (Table S1A). 1,20(OH)_2_D3 was less potent than 20(OH)D3 (p<0.05), however, more potent than pD, pL, and 7DHP (p<0.05), while 20(OH)pD and 20(OH)7DHP had no effect (Table S1A). The latter compounds were less efficient than 1,25(OH)_2_D3, 20,23(OH)_2_D3 and 20(OH)D3 (p<0.05 or 0.01) (Table S1A).

1,25(OH)_2_D3 and 20,23(OH)_2_D3 were the most effective in inhibiting proliferation of K562 human myeloid leukemia while slightly lower (p<0.05) but highly significant inhibition was seen for 20(OH)D3 (p<0.01) (Fig. 2B) (Table S1B). 1,20(OH)_2_D3 and pL were least potent, and 7DHP, 20(OH)7DHP and 20(OH)pD had no effect on these cells (Table S1B). Interestingly pD had a significant inhibitory effect on proliferation, which was not statistically different from the effects of 1,25(OH)_2_D3, 20,23(OH)_2_D3, 20(OH)D3 and 1,20(OH)_2_D3.

In human HL-60 promyelocytic and U937 myeloid leukemia all of the secosteroids and steroidal 5.7-dienes showed statistically significant inhibitory effects, however, their relative potency were different for both cell lines (Fig. 2C, D). The relative potency for HL-60 cells was as follows: 1,25(OH)_2_D3>20(OH)D3 = 20,23(OH)_2_D3 = 1,20(OH)_2_D3 = 20(OH)pD>pD = pL( = 7DHP) >20(OH)7DHP( = 7DHP), while for K562 cells it was 1,25(OH)_2_D3 >20(OH)D3 = 1,20(OH)_2_D3>20,23(OH)_2_D3 = pL = 7DHP = 20(OH)7DHP>pD = 20(OH)pD, respectively (Fig. 2C, D) (Table S1C, D).

#### Cell Differentiation

**Figure 3:**
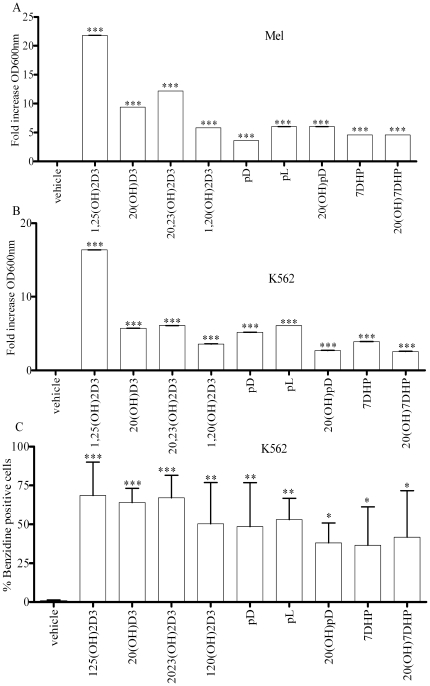
Novel secosteroids and steroidal 5.7-diene induce erythroid differentiation in leukemia cells. Induction of erythroid differentiation determined was from the the concentration of hemoglobin in an equal number of cells. Cells were treated for 7 days with different compounds (10^−7^ M), stained with benzidine solution and the amount of hemoglobin as hemin in Mel (**A**) or K562 (**B**) leukemia cells was determined by measuring the absorbance at 600 nm after lysis of the cells. ***p<0.001. **C**.The number of benzidine positive K562 cells after 7 days of treatment with different compounds (10^−7^ M). Cells were stained with benzidine solution and the number of benzidine positive (blue) cells was determined by counting 200 cells per microscopic field. The results are present as % of benzidine positive cells in comparison to total cell number. *p<0.05; **p<0.01; ***p<0.001.

The induction of erythroid differentiation was evident by the pink-red cell pellet of K562 cells after incubation for 7 days in the presence of secosteroids and steroidal 5.7-dienes (Fig. S2A). The benzidine staining shows that hemoglobin synthesis begins at day 2 (data not shown) and approximately 60%–80% of the cells treated with drugs for 7 days stained positively for benzidine (Fig. 3C; Fig. S2B). 1,25(OH)_2_D3, 20(OH)D3, 20,23(OH)_2_D3, 1,20(OH)_2_D3, pD and pL had similar potency for induction of differentiation as determined from the number of benzidine positive cells (Fig. 3; Table S2A). 20(OH)pD, 7DHP and 20(OH)7DHP were significantly less potent than 1,25(OH)_2_D3, 20(OH)D3 and 20,23(OH)_2_D3, but of similar in potency to other compounds tested with the exception of 1,20(OH)_2_D3 which was more potent than 7DHP (Table S2A).

When the level of differentiation was determined from the relative concentration of hemoglobin as hemin measured spectrophotometrically in an equal number of cells, all compounds tested induced significant cell differentiation. 1,25(OH)_2_D3 was more potent than other compounds with the sequence of potency being: 1,25(OH)_2_D3>20,23(OH)_2_D3> 20(OH)D3>pL>pD>1,20(OH)_2_D3>7DHP>20(OH)pD>20(OH)7DHP for K562 cells and 1,25(OH)_2_D3>20,23(OH)_2_D3>20(OH)D3>1,20(OH)_2_D3>pL = 20(OH)pD>7DHP = 20(OH)7DHP>pD in Mel cells (Fig. 3A and B; Table S2B, C).

**Figure 4 pone-0009907-g004:**
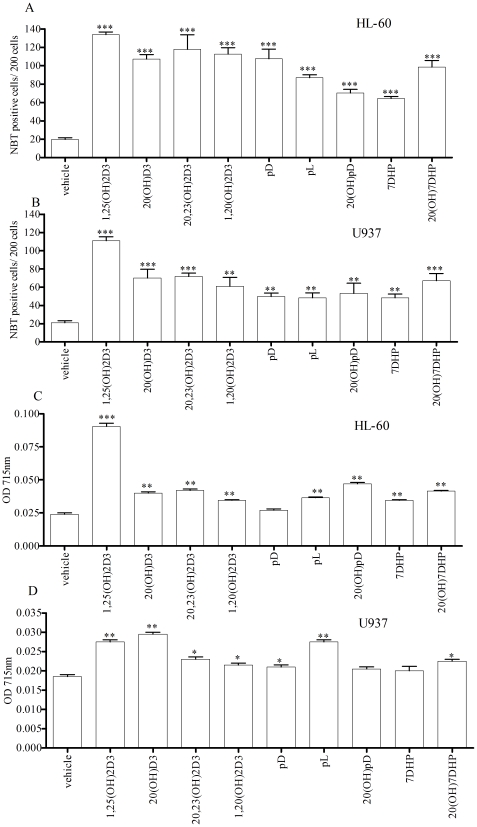
Novel secosteroids and steroidal 5.7-diene induce monocytic differentiation in HL-60 (A, C) and U937 cells (B, D). **A** and **B**. Cells were treated for 7 days with different compounds (10^−7^ M), stained with NBT and the number of NBT positive (blue) cells was determined counting 200 cells per field, and expressed as a % of the total cells. Induction of differentiation measured by the absorbance at 715 nm after lysis of the equal number of NBT stained cells is in **C** and **D**. *p<0.05; **p<0.01; ***p<0.001.

The induction of differentiation of HL-60 and U937 cells towards monocyte-like morphology was evaluated by NBT reduction and cell morphology. The first signs of morphological differentiation were observed at 72 h for all compounds tested with the differentiation being most pronounced for 1,25(OH)_2_D3 (data not shown). At day 5, the monocytic maturation pattern was similar for all compounds (Fig. S3) and by day 7 of treatment 82–88% of cells were NBT positive. In HL-60 cells 1,25(OH)_2_D3, 20,23(OH)_2_D3, 20(OH)D3, 1,20(OH)_2_D3, pL and pD showed similar potency in stimulation the formation of NBT positive cells. 20(OH)pD and 5,7-dienes were slightly less potent than vitamin D3 hydroxyderivatives on day 5 of treatment (Table S2D; Fig. S3). On Day 7, 1,25(OH)_2_D3, was equipotent to 20,23(OH)_2_D3 but was more efficient in induction of morphological differentiation than the other compounds tested (Fig. 4A; Table S3E). 20,23(OH)_2_D3 showed similar potency to 20(OH)D3, 1,20(OH)_2_D3, pL, pD and 20(OH)7DHP, while 20(OH)pD and 7DHP were less active (Fig. 4A; Table S2E). This was further confirmed when the absorbance of the supernatants from lysates was measured at 715 nm (Fig. 4C; Table S2F, G).

In U937 cells, all compounds tested induced cell differentiation by day 5 of treatment with 20,23(OH)_2_D3 being more potent than 1,20(OH)_2_D3, 7DHP, pL, pD and 20(OH)pD (figure not shown; Table S2H). On day 7, 1,25(OH)_2_D3 was the most potent compound, with 20,23(OH)_2_D3 being more potent than 7DHP, pL and pD and 20(OH)D3 being more potent than 7DHP (Fig. 4B, Table S2I). When the level of differentiation was measured spectrophotometrically to asses the relative effect on an equal number of cells, on day 5 of treatment all compounds induced differentiation with the exception of 7DHP and pL. 1,25(OH)_2_D3, 20(OH)D3 and pD were the most potent with 20(OH)D3 being significantly more potent than 1,20(OH)_2_D3, 7DHP, pL and 20(OH)7DHP (Table S2J). On day 7, the strongest induction of monocyte-like differentiation was observed for 1,25(OH)_2_D3, 20(OH)D3 and pL with other compounds being less potent, and 7DHP and 20(OH)pD having no effect (Fig. 4D, Table S2K).

### 4. Real time PCR and flow cytometry studies on HL-60 cells

**Figure 5 pone-0009907-g005:**
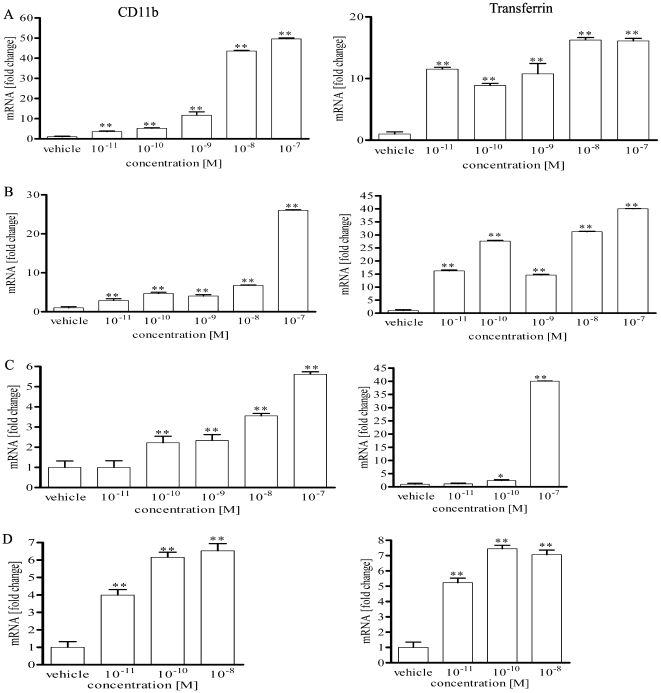
Novel secosteroidal and steroidal 5.7-diene induce the expression of differentiation genes in HL-60 cells. Real time PCR analysis of the expression of CD11b and transferrin genes in HL-60 cells. Cells were treated for 3 days with graded concentrations of 1,25(OH)_2_D3 (**A**), 20(OH)D3 (**B**), 20,23(OH)_2_D3 (**C**) and 1,20(OH)_2_D3 (**D**), mRNA was isolated and the gene expression analyzed. *p<0.05; **p<0.01; ***p<0.001 determined with one way ANOVA.

**Figure 6 pone-0009907-g006:**
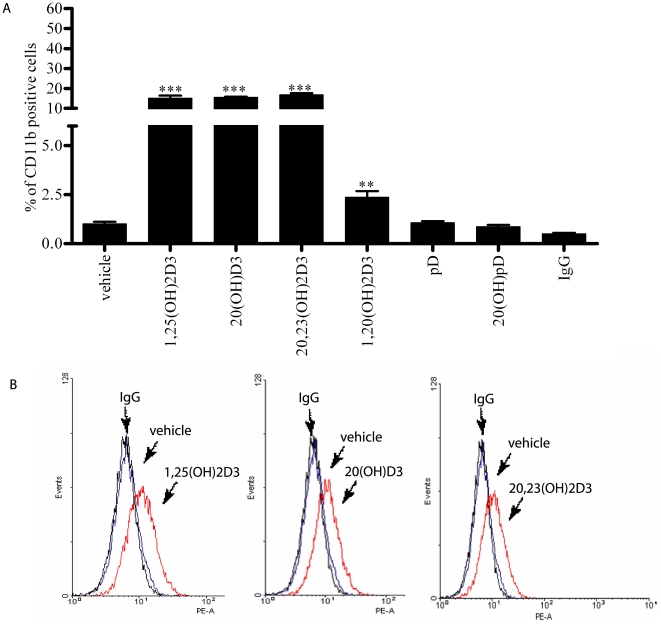
Novel secosteroids and steroidal 5.7-diene induce the expression of markers of differentiation in HL-60 cells. Flow cytometry analysis of CD11b protein expression in HL-60 cells. Cells were treated for 5 days with 10^−6^ M 1,25(OH)_2_D3 (**A**), 20(OH)D3 (**B**), 20,23(OH)_2_D3 (**C**), 1,20(OH)_2_D3 (**D**), pD (**E**) and 20(OH)pD (**F**), mRNA was isolated and the % of cells expressing CD11b protein determined. *p<0.05; **p<0.01; ***p<0.001.

Secosteroids with a full side chain (1,25(OH)_2_D3, 20,23(OH)_2_D3, 20(OH)D3, 1,20(OH)_2_D3) and those with just a two carbon side chain (pD and 20(OH)pD) were chosen for further studies on their antileukemic effects on HL-60 cells. Expression of CD11b and transferin genes was induced significantly and in a dose dependent manner only for secosteroids with a full-length side chain (1,25(OH)_2_D3, 20,23(OH)_2_D3, 20(OH)D3 and 1,20(OH)_2_D3) (Fig. 5), while pD and 20(OH)pD3 had no or minimal effects (not shown). These effects on CD11b were confirmed at the protein level by flow cytometry where 1,25(OH)_2_D3, 20,23(OH)_2_D3 and 20(OH)D3 were the strongest inducers of CD11b expression with 1,20(OH)_2_D3 being less potent and pD and 20(OH)pD3 having no effect (Fig. 6). Interestingly, among tested compounds only 1,25(OH)_2_D3 and 1,20(OH)_2_D3 stimulated CYP24 gene expression, with 1,20(OH)_2_D3 showing comparatively low potency (not shown). For example, at concentration of 10^−8^M 1,25(OH)_2_D3 stimulated CYP24 expression 700 times, while 1,20(OH)_2_D3 stimulated it only 8 times (not shown).

**Figure 7:**
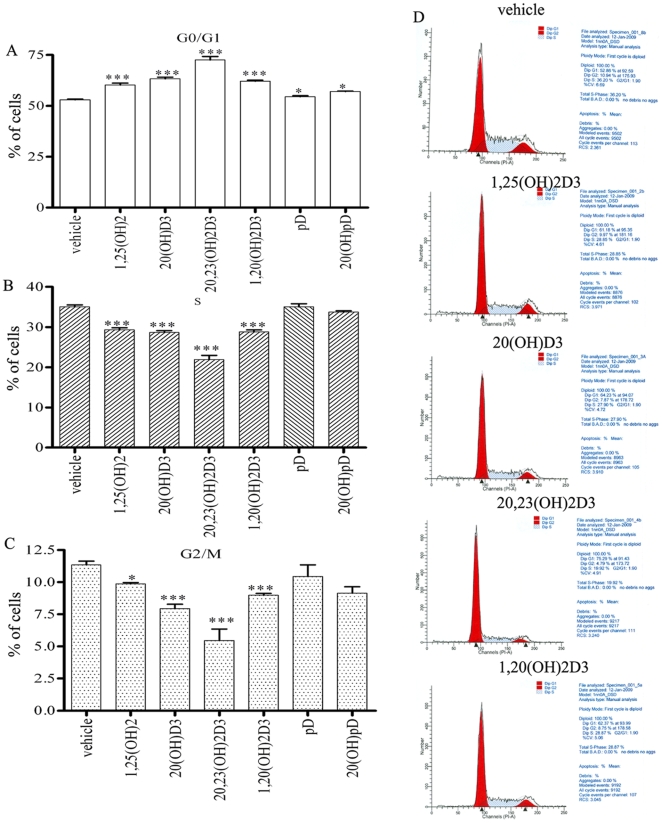
Novel secosteroids and steroidal 5.7-diene arrest HL-60 cells in G1/G0 phase of the cell cycle. Cell cycle analysis of the HL-60 human promyelocytic human leukemia shows arrest in G1/G0 phase (**A**). Statistically significant inhibition of S (**B**) and G2/M (**C**) phases is also evident for 1,25(OH)_2_D3, 20(OH)D3, 20,23(OH)_2_D3, 1,20(OH)2D3 but absent for pD and 20(OH)pD. Representative flow cytometry analysis for 20,23(OH)_2_D3 is in D. *p<0.05; **p<0.01; ***p<0.001.

Cell cycle analysis showed that secosteroids with a full-length side chain (1,25(OH)_2_D3, 20,23(OH)_2_D3, 20(OH)D3, 1,20(OH)_2_D3) induced highly significant G1/G0 phase arrest, while pD and 20(OH)pD were less potent (Fig. 7). Among the former, 20,23(OH)_2_D3 was the strongest inducer of arrest at G1/G0 and the strongest inhibitor of S and G2/M phases (p<0.05)(Fig. 7).

### 5. Molecular modeling studies

To understand the potential binding mode of 20*S*(OH)D3 in VDR, we selected the crystal structure of the VDR complex with calcitriol (PDB code: 1DB1)[35]and performed molecular modeling studies using Schrodinger Suite 2009 (Schrodinger Inc., New York, NY). The docking program successfully reproduced the original binding pose of the native ligand with an excellent docking score (−13.5, Supplemental Fig. S4). All of the six hydrogen bonding interactions between the ligand and the residues in VDR are reproduced in this validation: 3-OH to Ser274/Tyr143; 1-OH to Ser233/Arg270; and 25-OH to His393/HIP301.

Next we examined the potential binding of 20*S*(OH)D3 to the VDR. The 20*S*(OH) isomer (the metabolite generated by P450scc enzymes) overlaps with the native ligand very well with a docking score −11.4. We also performed docking calculations for a variety of D3 metabolites shown in Figure S1. The results are listed in Table S3. Overall, all the metabolites have good docking score, suggesting they may bind well to the VDR. In general, metabolites having 1 alfa-OH have better docking scores than those that do not have. Shorter chain analogs (pD3 analogs) generally have worse docking scores, suggesting that the removal of the side chain may be detrimental to the VDR binding. While we are fully aware of the limitations with molecular modeling, these docking studies suggest that there are high binding affinities of these compounds for the VDR, and the biological activity of these metabolites may still be generated via their interactions with the VDR.

## Discussion

Recently we have provided evidence that the mammalian P450scc system is capable of sequential hydroxylation of the side chain of vitamin D3 at position 20 and 23 [22], [23], [28] to metabolites that are biologically active in skin cells [26], [27], [34]. P450scc (in vitro and ex-vivo) can also transform 7DHC to 7DHP which can be further hydroxylated by classical steroidogenic enzymes to different steroidal 5,7-dienal hydroxy-products [29], [30]. These, if exposed to UVB, can change to secosteroidal products with only a 2-carbon side chain [30]–[32] some of which are biologically active in skin cells [29], [31]. In this study we show that novel secosteroids and 5,7-dienes, including 20,23(OH)_2_D3, 20(OH)D3, 1,20(OH)_2_D3, pD, pL, 20(OH)pD, 7DHP and 20(OH)7DHP, can inhibit proliferation and induce differentiation of MEL mouse erytholeukemia, human K562 chronic myeloid, HL-60 promyelocytic and U937 promonocytic leukemia cells, which is dependent on the cell type and the structure of the compound tested. These results are consistent with our separate studies showing that 20(OH)D3, 20,23(OH)_2_D3 and 1,20(OH)_2_D3 act in very similar manner to 1,25(OH)_2_D3 by inhibiting proliferation and inducing differentiation of cultured human keratinocytes [26], [28], [34].

In vivo data shown in Fig. 1 indicate that the calcemic effect of vitamin D3 derivatives can largely be separated from their other properties (inhibition of proliferation and stimulation of differentiation) as clearly demonstrated for 20(OH)D3. Interestingly, removal of the key hydroxyl group at C-1 whose presence is known to play a role in calcium regulation [5], [6], [20], has only a slight or no influence on the induction of differentiation of monocytes and leukemia cells (20,23(OH)_2_D3, 20(OH)D3, 1,20(OH)_2_D3 act in very similar manner (see figures 3–7). Interestingly, all of the secosteroids we tested had no significant effect on the induction of CYP24 expression, or a very small effect in the case of 1,20(OH)_2_D3, compared to the large stimulation by 1,25(OH)_2_D3. Again, this observation is in full agreement with our studies on human keratinocytes, where 20(OH)D3 and 20,23(OH)_2_D3 have shown poor induction of CYP24, while having equipotent activity with 1,25(OH)_2_D3 in the regulation of cellular phenotype [26], [34]. Importantly, gene silencing technology used on human keratinocytes has clearly demonstrated that the phenotypic action of 20(OH)D3 and 20,23(OH)_2_D3 are mediated through activation of VDR [26], [27], [34]. Therefore, because of this intriguing dissociation between phenotypic effects seen in leukemia cells (present studies) and keratinocytes (parallel studies) we have examined the potential binding of 20(OH)D3 to the VDR (figure S4). We have found that the 20*S*(OH) isomer (the metabolite generated by P450scc [22], [23]) overlaps with the native ligand (calcitriol) very well having comparable docking score (figure S4). This opens a new and exciting area to study on the correlation between structure and activity of P450scc and CYP27A1 and CYP27B1 generated active vitamin D3 hydroxyderivatives Since these compounds also differ in the presence or absence of a hydroxyl group at C-25, we suggest that a C-25 hydroxyl group, in addition to C-1 (see above), is necessary for strong induction of CYP24 gene expression. Future studies on this subject are mandatory, since CYP24 is the major enzyme causing inactivation of vitamin D, and respectively slower rates of inactivation could be predicted for the P450scc-derived secosteroids, which should be of advantage for therapeutic use of these compounds.

The observed activities of the various secosteroids and steroidal 5–7dienes we tested were influenced by the genetic background of the cell lines used, suggesting the existence of cell-type specific factors that can modulate activities. Such factors might include the cocktail of transcription factors present, and the concentration of vitamin D metabolizing enzymes in the cells such as CYP24, CYP27A1 and CYP27B1. Despite this variability between cell types, the non-calcemic 20(OH)D3 and its hydroxyl-derivatives generally exerted effects on cell proliferation and differentiation comparable to those of 1,25(OH)_2_D3. Furthermore we observed functional similarity with the effects induced by 20(OH)D3 and 20,23(OH)_2_D3 in epidermal keratinocytes, e.g., G1/G0 arrest connected with induction of their differentiation program [26], [34]. The shortening of the side chain resulted in decreased activity, which is in agreement with lower docking score in comparison to 1,25(OH)_2_D3 (Table S3). Nevertheless, these compounds, pD3 and 20-OH pD3, do induce leukemia cell differentiation, similarly as in melanoma cells [29], [31]. This suggests that the mechanism of action of the tested compounds is conserved among different cell types including leukemias (cell of hematopoietic origin), normal skin keratinocytes [26], [34] and malignant melanoma cells [29], [31].

Acute myeloid leukemias (AML) are high grade malignancies originating from blood cell precursors in bone marrow. Depending on the lineage of leukemic cells they can be divided into myeloid, monocytic, erythroid and megakaryocytic. AML is characterized by accumulation of immature forms (blasts) in bone marrow and subsequently in peripheral blood and are rapidly and universally fatal if not treated. Developed over the last three decades, chemotherapeutic regimens to treat AML markedly improved the rate of remission and survival in AML patients. However, many leukemias show primary resistance to current therapy and many patients suffers from AML relapse following initial remission. Relapsed disease often becomes resistant to currently available chemotherapeutic drugs and short of bone marrow transplant such relapsed patients become incurable and die. These findings necessitate a continuing search for new drugs or adjuvant therapeutic approaches to treat AML.It is well established that hematopoietic cells and their malignant counterparts (leukemic blasts) express VDR. Moreover, in vitro studies of myeloid leukemia cells show that there is strong response to the active form of vitamin D ranging from induction of differentiation to induction of apoptosis or autophagy, with resulting attenuation of proliferation [10], [12]–[14], [36]. Unfortunately in vivo use of these promising drugs is limited by their toxic (calcemic) activity. In this study we report that newly identified and characterized derivatives of vitamin D that have low or no calcemic activity still retain their potent anti-leukemic effects. Furthermore, the above compounds have the potential to be normally generated in skin (from their 5,7-dienes precursors) exposed to solar radiation [29]–[31], [37]. Potentially these derivatives can be synthesized in other tissues in the body expressing P450scc, for example the adrenal gland [22], [23], [25], [30], [38]. Interestingly the number of tissues in which expression of P450scc has been identified is growing and includes bone, thymus, skin and brain as examples [30], [39]–[44], raising the possibility of local production of the ligands identified in this study in the bone marrow environment. Therefore, we believe that we have identified a family of non-toxic and physiological compounds that can be used in therapy of acute leukemias [22], [23], [28]–[31].

In summary, novel secosteroidal products of P450scc metabolism of vitamin D3 and pro-vitamin D3, show excellent anti-leukemia activity in vitro with 20(OH)D3 deserving special attention because of its relatively high potency and lack of calcemic activity. Moreover, since these compounds potentially can be synthesized in vivo in adrenal gland or generated by skin and theoretically may be produced by many other tissues including bone, further studies are required to define their potential physiological and pathophysiological role in bone marrow homeostasis and leukemiagenesis.

## Materials and Methods

### 1. Steroids and secosteroids

Chemical structures of the compounds tested are shown in Figure S1. 1,25(OH)_2_D3 was purchased from (Fluka). 20(OH)D3 and 20,23(OH)_2_D3 were biochemically synthesized as described previously using an in vitro reconstituted P450scc system with vitamin D3 (Sigma) as substrate [23], [30]. 1,20(OH)_2_D3 was biochemically synthesized from 1α-hydroxyvitamin D3 (Sigma) as described previously [28].The products were purified by TLC followed by RP-HPLC and identities were confirmed based on mass and UV spectra, as well as on retention times in comparison to standards previously characterized by NMR [23], [28], [30]. The compounds were aliquoted, dried and stored at −80°C until use.

7DHP and 20(OH)7DHP were synthesized and purified as described previously [31], [32]. After NMR confirmation of their structures, the steroids were purified by HPLC, aliquoted and stored for further use or subjected to photochemical transformation using a Biorad UV Transilluminator 2000 (Biorad, Hercules, CA). Spectral characteristics of the UVB (280–320 nm) source were published previously [45], [46] and its strength (4.8±0.2 mW cm^−2^) was routinely measured with a digital UVB Meter Model 6.0 (Solartech Inc., Harrison Twp, MI). Irradiation was followed by incubation for 14 h at room temperature then selected products were purified by RP-HPLC chromatography as described [31], [32]. The major products were identified on the basis of their retention time and characteristic UV absorption. Initial identification was confirmed by means of MS and NMR measurements [31], [32]. The resulting secosteroids including pregnacalciferol (pD), pregnalumisterol (pL) and 20-hydroxy-pregnacalciferol (20(OH)pD) were RP-HPLC purified, aliquoted and stored at −80°C [31], [32].

### 2. Testing on leukemia cell lines

MEL mouse erythroleukemia [47], human K562 chronic myeloid leukemia (purchased from American Tissue Culture Collection, USA), and human HL-60 human promyelocytic [48] and U937 promonocytic leukemia [49] cells were cultured in RPMI 1640 containing 10% fetal bovine serum (FBS) (Atlanta Biologics)) and 1% penicillin/streptomycin/amphotericin antibiotic solution (Sigma) at 37°C in 5% CO_2_. Test compounds were dissolved in ethanol and added to the cultures to reach final concentrations as listed. Ethanol at a concentration of 0.1% was used as a vehicle (negative) control, while 2% DMSO or 32 nM TPA served as positive controls for studies on cell differentiation. U937 and MEL cell lines were generous gift from Dr S. Hanissian (University of Tennessee HSC) and HL-60 from Dr M. Radic (University of Tennessee HSC).

#### Cell proliferation

Cells at concentrations of 2×10^6^ cells/ml (K56 and MEL lines) and 10^7^ cells/ml (HL-60 and U937 lines) were inoculated into T25 flasks (TPP, Midwest Scientific) containing 10 ml of RPMI supplemented with charcoal treated 10% FBS. The test compounds were added at concentrations of 10^−7^ M every day with media being changed every 72 hours. After 7 days days the cells were stained in 0.4% trypan blue (Sigma) and the viable cells counted with a hemacytometer.

#### Cell cycle analysis

Cell cycle analysis was performed by flow cytometry following standard protocols used in our laboratories [26], [50]. Briefly, HL-60 cells were cultured in RPMI 1640 plus charcoal treated 10% FBS (HyClone). Test compounds were added to a final concentration 10^−6^ M in 0.1% ethanol every day with media being changed every third day. On day five the cells were washed in PBS, fixed in 70% cold ethanol and stained with propidium iodine (Sigma). DNA content analysis was performed with a FACS Calibur flow cytometer.

#### Cell differentiation

To estimate erythroid differentiation (production of hemoglobin), first we evaluated the number of benzidine positive cells after 5 and 7 days in culture. Cells were centrifuged and washed four times with PBS and resuspended in 1 ml of fresh PBS. For hemoglobin determination, a benzidine staining solution was freshly prepared by mixing one part of 30% hydrogen peroxide, one part of base stock solution of 3% benzidine in 90% acetic acid, and 5 parts of water. The solution was diluted 1∶10 with the cell suspension and 250 µl aliquots added to 4 wells of a 24-well plate. After 10 min of incubation at room temperature, benzidine-positive cells were counted under the microscope with a minimum of 200 cells scored. Second, to define the relative content of hemoglobin spectrophotometrically [51], equal number of cells (7×10^6^) were washed with cold PBS and lysed for 20 min in 100 µl of lysis buffer (0.2% Triton X-100 in 100 mM potassium phosphate buffer, pH 7.8). The lysates were centrifuged for 15 min at 1500 r.p.m. and 100 µl of the supernatant was incubated with 2 ml of benzidine solution (5 mg/ml in glacial acetic acid) and 2 ml 30% H_2_O_2_ for 10 min at room temperature in the dark. Absorption was measured at 600 nm. Data are shown as fold increases in comparison to the level of hemoglobin in vehicle-treated cells.

Differentiation of HL-60 or U937 cells toward monocytes-like morphology and NBT-reduction was assessed after 5 and 7 days. Cells (2×10^6^) were washed with PBS four times and resuspended in 200 µl of NBT solution (4 mg/ml). After the addition of 200 µl of TPA solution (2 µg/ml in PBS) cells were incubated at 37°C for 60 min in 24-well plates. Cell differentiation was assessed by intracellular blue formazan deposits. The NBT positive and negative cells were scored under light microscopy examination (20×) with a minimum of 200 cells scored. [52]. For spectrophotometric analysis the cells were washed twice with buffer containing cold bovine serum albumin solution (17 mg/ml BSA, 137 mM NaCl, 5 mM KCl, 0.8 mM MgSO_4_, 10 mM, HEPES, pH 7.4) to remove unreacted NBT, and the insoluble formazan deposits in the resulting pellets were solubilized in 1 ml of 90% DMSO, 0.1% SDS and 0.01 mM NaOH by vigorous vortexing. The samples were centrifuged 5 min at 1,500 x g to remove the cellular debris, and then the absorbance of supernatants measured at 715 nm. Data are expressed as change in A_715_/10^6^ cells [53].

#### CD11b expression

Staining of the CD11b cell differentiation marker was carried out according to the manufacturer’s protocol as follows. Cultured cells were resuspended in cold wash buffer (PBS supplemented with 1% FBS and 1% human serum to block non-specific F_c_-mediated binding) at a concentration of 2×10^6^/ml. After centrifugation, the cell pellet was resuspended in 100 µl wash buffer. Aliquots (50 µl) of this were incubated with 50 µl of diluted antibody, either phycoerythrin-conjugated mouse anti-human CD11b antibody or phycoerythrin-conjugated mouse IgG isotype as control (both from BD Pharmingen, San Diego, California). After 30 min the cells were centrifuged at at 400 x g and resuspended in 1 ml of wash buffer. This step was repeated with cells finally being resuspended in 200 µl of wash buffer for flow cytometry [54].

#### Real-time RT PCR

The RNA from HL-60 cells treated as described above was isolated using the Absolutely RNA Miniprep Kit (Stratagene, La Jolla, CA). Reverse transcription (1 µg RNA/reaction) was performed using the Transcriptor First Strand cDNA Synthesis Kit (Roche, Mannheim, Germany). Real-time PCR was performed using undiluted cDNA and a TaqMan PCR Master Mix. Reactions (in triplicate) were performed at 50°C for 2 min, 95°C for 10 min and then 50 cycles of 95°C for 15 sec, 60°C for 30 sec and 72°C for 30 sec. The primers and probes were designed with the universal probe library (Roche). Data were collected on a Roche Light Cycler 480. The amount of amplified product for each gene was compared to that for β actin using a comparative C_T_ method. A list of the primers used for RT-PCR DNA amplification is shown in Table S4.

### 3. Testing of calcemic effect

The calcemic effects of 20(OH)D3 and 1,20(OH)_2_D3 were compared with 25(OH)D3 and 1,25(OH)_2_D3. Briefly, weanling rats were obtained from Holtzman and fed a vitamin D deficient diet for 3 months before they were divided into 13 groups (6 animals per group) and injected with either vehicle (propylene glycol) or 3 concentrations (0.3 µg/kg, 1.0 µg/kg, 3.0 µg/kg) of 25(OH)D3, 1,25(OH)_2_D3, 20(OH)D3 or 1,20(OH)_2_D3 dissolved in propylene glycol for 7 consecutive days. A day after the final dosing, blood was obtained by heart puncture and serum was prepared. Serum calcium was determined by using a kit (DICA-500 QuantiChrom™) from BioAssay Systems (Hayward, CA). Data were analyzed using a student's 1-tailed independent t-test with a p value indicating significance at p<0.05.

### 4. Statistical analysis

Data were analyzed with GraphPad Prizm Version 4.0 (GraphPad Software Inc., San Diego, CA, USA) using the t-test or one way ANOVA with appropriate post-hoc tests. Differences were considered significant when p<0.05. The data are presented as means −/+ SE.

## Supporting Information

Figure S1 Structures and origin of novel secosteroids. Arrows show direction of enzymatic reactions or photochemical transformation. 20HSD: 20-hydroxysteroid dehydrogenase; P40scc: CYP11A1; 1-alpha hydroxylase: CYP27B1.
(0.74 MB TIF)

Figure S2 Morphological aspects of erythroid differentiation in K562 human leukemia after 7 days of treatment with 10^−7^M of listed compounds. A. Representative cell pellets after 7 days of treatment with 1,25(OH)_2_D3 and 20,23(OH)_2_D3. B. Representative microscopic fields showing benzidine positive K562 cells. The cells were stained with benzidine solution and photographed in light microscopy, 20× magnification.
(0.14 MB PDF)

Figure S3 Morphological aspects of monocytic differentiation in HL-60 (A) and U932 (B) human leukemia cells after 5 days of treatment with 10^−7^M of listed compounds. The cells were stained with NBT solution (blue) and photographed in light microscopy, 20× magnification.
(0.22 MB PDF)

Figure S4 Superimposition of 20S(OH)D3 (dark green, docking score −11.4) and the native ligands in VDR (docking score −13.5). Yellow dotted lines shows that the docking program well reproduced the six hydrogen bonding interactions between the native ligand and VDR that are presented in the crystal structures.
(0.57 MB TIF)

Table S1 Student t test analysis of inhibitory effect of tested compounds on leukemia cells proliferation
(0.11 MB DOC)

Table S2 Student t test analysis of stimulatory effect of tested compounds on leukemia differentiation.
(0.26 MB DOC)

Table S3 Docking scores of the biding of tested ligands to the VDR.
(0.04 MB DOC)

Table S4 Primers used for real time RT-PCR analysis.
(0.03 MB DOC)
