# Supplementary figures and images for: Products of Vitamin D3 or 7-Dehydrocholesterol Metabolism by Cytochrome P450scc Show Anti-Leukemia Effects, Having Low or Absent Calcemic Activity
Source: PLoS One. 2010 Mar 26;5(3):e9907. doi: 10.1371/journal.pone.0009907 (PMC2845617; doi:10.1371/journal.pone.0009907)

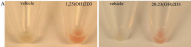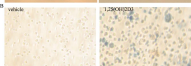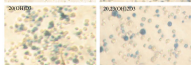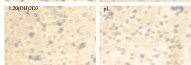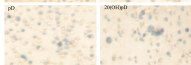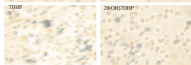

Supplement: Figure S2 — Morphological aspects of erythroid differentiation in K562 human leukemia after 7 days of treatment with 10−7M of listed compounds. A. Representative cell pellets after 7 days of treatment with 1,25(OH)2D3 and 20,23(OH)2D3. B. Representative microscopic fields showing benzidine positive K562 cells. The cells were stained with benzidine solution and photographed in light microscopy, 20× magnification. (0.14 MB PDF) [file pone.0009907.s002.pdf]

A vehicle

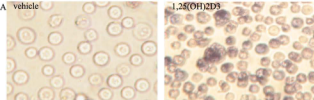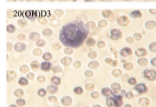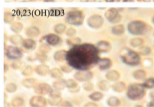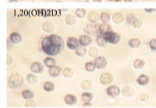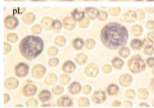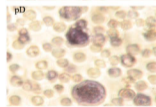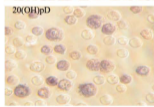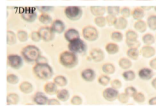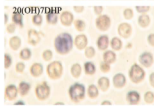

B vehicle

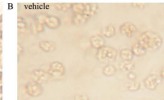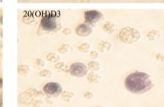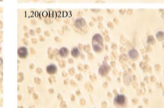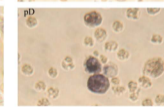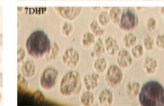

1,25(OH)2D3

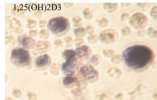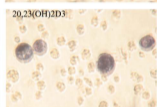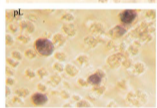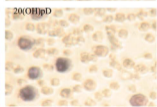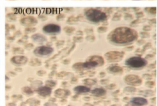

Supplement: Figure S3 — Morphological aspects of monocytic differentiation in HL-60 (A) and U932 (B) human leukemia cells after 5 days of treatment with 10−7M of listed compounds. The cells were stained with NBT solution (blue) and photographed in light microscopy, 20× magnification. (0.22 MB PDF) [file pone.0009907.s003.pdf]

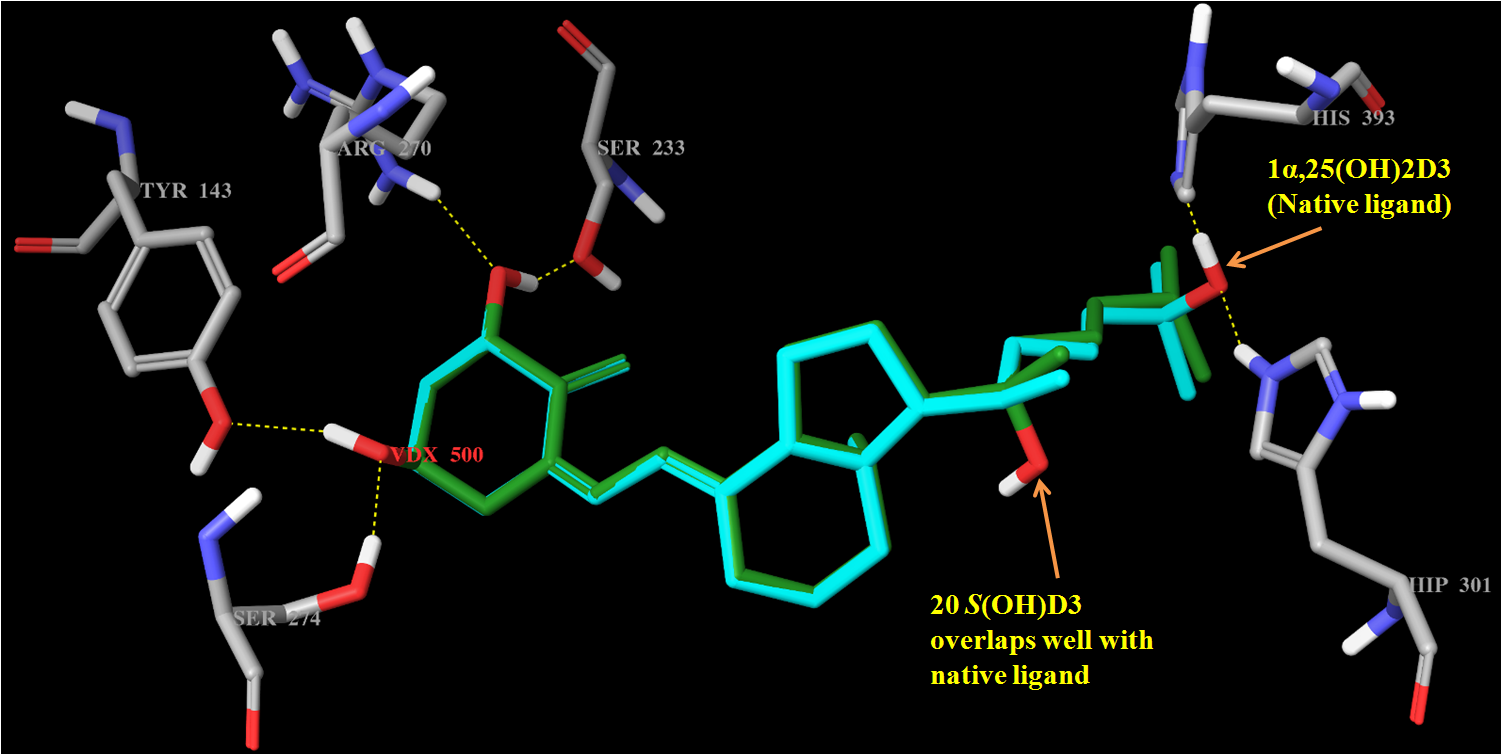

Supplement: Figure S4 — Superimposition of 20S(OH)D3 (dark green, docking score −11.4) and the native ligands in VDR (docking score −13.5). Yellow dotted lines shows that the docking program well reproduced the six hydrogen bonding interactions between the native ligand and VDR that are presented in the crystal structures. (0.57 MB TIF) [file pone.0009907.s004.tif]
