# Supplementary material for: Products of Vitamin D3 or 7-Dehydrocholesterol Metabolism by Cytochrome P450scc Show Anti-Leukemia Effects, Having Low or Absent Calcemic Activity
Source: PLoS One. 2010 Mar 26;5(3):e9907. doi: 10.1371/journal.pone.0009907 (PMC2845617; doi:10.1371/journal.pone.0009907)
Supplement: Table S1 — Student t test analysis of inhibitory effect of tested compounds on leukemia cells proliferation (0.11 MB DOC) [file pone.0009907.s005.doc]

**Table S1.** Student t test analysis of inhibitory effect of tested compounds on leukemia cells proliferation

A. Mouse Mel leukemia

|  | **vehicle** | **1,25**  **(OH)2D3** | **20(OH)**  **D3** | **20,23**  **(OH)2D3** | **1,20**  **(OH)2D3** | **pD** | **pL** | **20(OH)**  **pD** | **7DHP** | **20(OH)**  **7DHP** |
| --- | --- | --- | --- | --- | --- | --- | --- | --- | --- | --- |
| **vehicle** |  | P<0.01 | P<0.01 | P<0.01 | P<0.01 | P<0.05 | P<0.05 | P>0.05 | P<0.05 | P>0.05 |
| **1,25(OH)2D3** | P<0.01 |  | P<0.05 | P>0.05 | P<0.01 | P<0.01 | P<0.01 | P<0.01 | P<0.01 | P<0.01 |
| **20(OH)D3** | P<0.01 | P<0.05 |  | P<0.05 | P<0.05 | P<0.05 | P<0.05 | P<0.01 | P<0.01 | P<0.01 |
| **20,23(OH)2D3** | P<0.01 | P>0.05 | P<0.05 |  | P<0.01 | P<0.01 | P<0.01 | P<0.01 | P<0.01 | P<0.01 |
| **1,20(OH)2D3** | P<0.01 | P<0.01 | P<0.05 | P<0.01 |  | P<0.05 | P<0.05 | P<0.05 | P<0.05 | P<0.05 |
| **pD** | P<0.05 | P<0.01 | P<0.05 | P<0.01 | P<0.05 |  | P>0.05 | P>0.05 | P>0.05 | P>0.05 |
| **pL** | P<0.05 | P<0.01 | P<0.05 | P<0.01 | P<0.05 | P>0.05 |  | P>0.05 | P>0.05 | P>0.05 |
| **20(OH)pD** | P>0.05 | P<0.01 | P<0.01 | P<0.01 | P<0.05 | P>0.05 | P>0.05 |  | P>0.05 | P>0.05 |
| **7DHP** | P<0.05 | P<0.01 | P<0.01 | P<0.01 | P<0.05 | P>0.05 | P>0.05 | P>0.05 |  | P>0.05 |
| **20(OH)7DHP** | P>0.05 | P<0.01 | P<0.01 | P<0.01 | P<0.05 | P>0.05 | P>0.05 | P>0.05 | P>0.05 |  |

**B.** **Human K562 leukemia**

|  | **vehicle** | **1,25**  **(OH)2D3** | **20(OH)**  **D3** | **20,23**  **(OH)2D3** | **1,20**  **(OH)2D3** | **pD** | **pL** | **20(OH)**  **pD** | **7DHP** | **20(OH)**  **7DHP** |
| --- | --- | --- | --- | --- | --- | --- | --- | --- | --- | --- |
| **vehicle** |  | P<0.01 | P<0.01 | P<0.01 | P<0.01 | P<0.05 | P<0.05 | P>0.05 | P>0.05 | P>0.05 |
| **1,25(OH)2D3** | P<0.01 |  | P<0.01 | P>0.05 | P<0.01 | P>0.05 | P<0.05 | P<0.01 | P<0.01 | P<0.05 |
| **20(OH)D3** | P<0.01 | P<0.01 |  | P<0.01 | P<0.01 | P>0.05 | P<0.05 | P<0.01 | P<0.01 | P>0.05 |
| **20,23(OH)2D3** | P<0.01 | P>0.05 | P<0.01 |  | P<0.01 | P>0.05 | P<0.05 | P<0.01 | P<0.01 | P<0.05 |
| **1,20(OH)2D3** | P<0.01 | P<0.01 | P<0.01 | P<0.01 |  | P>0.05 | P>0.05 | P>0.05 | p>0.05 | p>0.05 |
| **pD** | P<0.05 | p>0.05 | p>0.05 | p>0.05 | p>0.05 |  | p>0.05 | p>0.05 | p>0.05 | p>0.05 |
| **pL** | P<0.05 | P<0.05 | P<0.05 | P<0.05 | p>0.05 | p>0.05 |  | p>0.05 | p>0.05 | p>0.05 |
| **20(OH)pD** | p>0.05 | P<0.01 | P<0.01 | P<0.01 | p>0.05 | p>0.05 | p>0.05 |  | p>0.05 | p>0.05 |
| **7DHP** | p>0.05 | P<0.01 | P<0.01 | P<0.01 | p>0.05 | p>0.05 | p>0.05 | p>0.05 |  | p>0.05 |
| **20(OH)7DHP** | p>0.05 | P<0.05 | p>0.05 | P<0.05 | p>0.05 | p>0.05 | p>0.05 | p>0.05 | p>0.05 |  |

**C.** Human HL-60 leukemia

|  | **vehicle** | **1,25**  **(OH)2D3** | **20(OH)**  **D3** | **20,23**  **(OH)2D3** | **1,20**  **(OH)2D3** | **pD** | **pL** | **20(OH)**  **pD** | **7DHP** | **20(OH)**  **7DHP** |
| --- | --- | --- | --- | --- | --- | --- | --- | --- | --- | --- |
| **vehicle** |  | P<0.001 | P<0.001 | P<0.001 | P<0.001 | P<0.01 | P<0.001 | P<0.001 | P<0.001 | P<0.001 |
| **1,25(OH)2D3** | P<0.001 |  | P<0.01 | P<0.01 | P<0.01 | P<0.001 | P<0.001 | P<0.01 | P<0.001 | P<0.001 |
| **20(OH)D3** | P<0.001 | P<0.01 |  | p>0.05 | p>0.05 | P<0.01 | P<0.01 | p>0.05 | P<0.01 | P<0.01 |
| **20,23(OH)2D3** | P<0.001 | P<0.01 | p>0.05 |  | p>0.05 | P<0.01 | P<0.01 | p>0.05 | P<0.01 | P<0.01 |
| **1,20(OH)2D3** | P<0.001 | P<0.01 | p>0.05 | p>0.05 |  | P<0.01 | P<0.01 | p>0.05 | P<0.01 | P<0.01 |
| **pD3** | P<0.01 | P<0.001 | P<0.01 | P<0.01 | P<0.01 |  | p>0.05 | P<0.05 | p>0.05 | P<0.05 |
| **pL3** | P<0.001 | P<0.001 | P<0.01 | P<0.01 | P<0.01 | p>0.05 |  | P<0.05 | p>0.05 | P<0.05 |
| **20(OH)pD3** | P<0.001 | P<0.01 | p>0.05 | p>0.05 | p>0.05 | P<0.05 | P<0.05 |  | P<0.05 | P<0.05 |
| **7DHP** | P<0.001 | P<0.001 | P<0.01 | P<0.01 | P<0.01 | p>0.05 | p>0.05 | P<0.05 |  | p>0.05 |
| **20(OH)7DHP** | P<0.001 | P<0.001 | P<0.01 | P<0.01 | P<0.01 | P<0.05 | P<0.05 | P<0.05 | p>0.05 |  |

D. Human U937 leukemia

|  | **vehicle** | **1,25**  **(OH)2D3** | **20(OH)**  **D3** | **20,23**  **(OH)2D3** | **1,20**  **(OH)2D3** | **pD** | **pL** | **20(OH)**  **pD** | **7DHP** | **20(OH)**  **7DHP** |
| --- | --- | --- | --- | --- | --- | --- | --- | --- | --- | --- |
| **vehicle** |  | P<0.001 | P<0.001 | P<0.01 | P<0.001 | P<0.05 | P<0.01 | P<0.05 | P<0.01 | P<0.01 |
| **1,25(OH)2D3** | P<0.001 |  | P<0.05 | P<0.01 | P<0.05 | P<0.001 | P<0.01 | P<0.001 | P<0.01 | P<0.001 |
| **20(OH)D3** | P<0.001 | P<0.05 |  | P<0.05 | p>0.05 | P<0.01 | P<0.01 | P<0.01 | P<0.01 | P<0.01 |
| **20,23(OH)2D3** | P<0.01 | P<0.01 | P<0.05 |  | P<0.05 | P<0.05 | p>0.05 | P<0.05 | p>0.05 | p>0.05 |
| **1,20(OH)2D3** | P<0.001 | P<0.05 | p>0.05 | P<0.05 |  | P<0.01 | P<0.05 | P<0.01 | P<0.01 | P<0.01 |
| **pD3** | P<0.05 | P<0.001 | P<0.01 | P<0.05 | P<0.01 |  | P<0.01 | p>0.05 | P<0.01 | P<0.05 |
| **pL3** | P<0.01 | P<0.01 | P<0.01 | p>0.05 | P<0.05 | P<0.01 |  | P<0.01 | P<0.05 | P<0.01 |
| **20(OH)pD3** | P<0.05 | P<0.001 | P<0.01 | P<0.05 | P<0.01 | p>0.05 | P<0.01 |  | P<0.01 | P<0.05 |
| **7DHP** | P<0.01 | P<0.01 | P<0.01 | p>0.05 | P<0.01 | P<0.01 | P<0.05 | P<0.01 |  | P<0.05 |
| **20(OH)7DHP** | P<0.01 | P<0.001 | P<0.01 | p>0.05 | P<0.01 | P<0.05 | P<0.01 | P<0.05 | P<0.05 |  |
