# Supplementary material for: Products of Vitamin D3 or 7-Dehydrocholesterol Metabolism by Cytochrome P450scc Show Anti-Leukemia Effects, Having Low or Absent Calcemic Activity
Source: PLoS One. 2010 Mar 26;5(3):e9907. doi: 10.1371/journal.pone.0009907 (PMC2845617; doi:10.1371/journal.pone.0009907)
Supplement: Table S2 — Student t test analysis of stimulatory effect of tested compounds on leukemia differentiation. (0.26 MB DOC) [file pone.0009907.s006.doc]

**Table S2.** Student t test analysis of stimulatory effect of tested compounds on leukemia differentiation

**A.** **Human K562 leukemia: number of benzidine positive cells**

|  | **vehicle** | **1,25**  **(OH)2D3** | **20(OH)**  **D3** | **20,23**  **(OH)2D3** | **1,20**  **(OH)2D3** | **pD** | **pL** | **20(OH)**  **pD** | **7DHP** | **20(OH)**  **7DHP** |
| --- | --- | --- | --- | --- | --- | --- | --- | --- | --- | --- |
| **vehicle** |  | P<0.001 | P<0.001 | P<0.001 | P<0.01 | P<0.01 | P<0.01 | P<0.05 | P<0.05 | P<0.05 |
| **1,25(OH)2D3** | P<0.001 |  | p>0.05 | p>0.05 | p>0.05 | p>0.05 | p>0.05 | P<0.05 | P<0.05 | P<0.05 |
| **20(OH)D3** | P<0.001 | p>0.05 |  | p>0.05 | p>0.05 | p>0.05 | p>0.05 | P<0.05 | P<0.05 | P<0.05 |
| **20,23(OH)2D3** | P<0.001 | p>0.05 | p>0.05 |  | p>0.05 | p>0.05 | p>0.05 | P<0.05 | P<0.05 | P<0.05 |
| **1,20(OH)2D3** | P<0.01 | p>0.05 | p>0.05 | p>0.05 |  | p>0.05 | p>0.05 | p>0.05 | P<0.05 | p>0.05 |
| **pD** | P<0.01 | p>0.05 | p>0.05 | p>0.05 | p>0.05 |  | p>0.05 | p>0.05 | p>0.05 | p>0.05 |
| **pL** | P<0.01 | p>0.05 | p>0.05 | p>0.05 | p>0.05 | p>0.05 |  | p>0.05 | p>0.05 | p>0.05 |
| **20(OH)pD** | P<0.05 | P<0.05 | P<0.05 | P<0.05 | p>0.05 | p>0.05 | p>0.05 |  | p>0.05 | p>0.05 |
| **7DHP** | P<0.05 | P<0.05 | P<0.05 | P<0.05 | P<0.05 | p>0.05 | p>0.05 | p>0.05 |  | p>0.05 |
| **20(OH)7DHP** | P<0.05 | P<0.05 | P<0.05 | P<0.05 | p>0.05 | p>0.05 | p>0.05 | p>0.05 | p>0.05 |  |

**B.** **Human K562 leukemia: amount of hemoglobin measured spectrophotometrically**

|  | **vehicle** | **1,25**  **(OH)2D3** | **20(OH)**  **D3** | **20,23**  **(OH)2D3** | **1,20**  **(OH)2D3** | **pD** | **pL** | **20(OH)**  **pD** | **7DHP** | **20(OH)**  **7DHP** |
| --- | --- | --- | --- | --- | --- | --- | --- | --- | --- | --- |
| **vehicle** |  | P<0.001 | P<0.001 | P<0.001 | P<0.001 | P<0.001 | P<0.001 | P<0.001 | P<0.001 | P<0.001 |
| **1,25(OH)2D3** | P<0.001 |  | P<0.001 | P<0.001 | P<0.001 | P<0.001 | P<0.001 | P<0.001 | P<0.001 | P<0.001 |
| **20(OH)D3** | P<0.001 | P<0.001 |  | P<0.001 | P<0.001 | P<0.001 | P<0.001 | P<0.001 | P<0.001 | P<0.001 |
| **20,23(OH)2D3** | P<0.001 | P<0.001 | P<0.001 |  | P<0.001 | P<0.001 | P<0.05 | P<0.001 | P<0.001 | P<0.001 |
| **1,20(OH)2D3** | P<0.001 | P<0.001 | P<0.001 | P<0.001 |  | P<0.001 | P<0.001 | P<0.001 | P<0.001 | P<0.001 |
| **pD** | P<0.001 | P<0.001 | P<0.001 | P<0.001 | P<0.001 |  | P<0.001 | P<0.001 | P<0.001 | P<0.001 |
| **pL** | P<0.001 | P<0.001 | P<0.001 | P<0.05 | P<0.001 | P<0.001 |  | P<0.001 | P<0.001 | P<0.001 |
| **20(OH)pD** | P<0.001 | P<0.001 | P<0.001 | P<0.001 | P<0.001 | P<0.001 | P<0.001 |  | P<0.001 | P<0.01 |
| **7DHP** | P<0.001 | P<0.001 | P<0.001 | P<0.001 | P<0.001 | P<0.001 | P<0.001 | P<0.001 |  | P<0.001 |
| **20(OH)7DHP** | P<0.001 | P<0.001 | P<0.001 | P<0.001 | P<0.001 | P<0.001 | P<0.001 | P<0.01 | P<0.001 |  |

C. Mouse Mel leukemia: amount of hemoglobin measured spectrophotometrically

|  | **vehicle** | **1,25**  **(OH)2D3** | **20(OH)**  **D3** | **20,23**  **(OH)2D3** | **1,20**  **(OH)2D3** | **pD** | **pL** | **20(OH)**  **pD** | **7DHP** | **20(OH)**  **7DHP** |
| --- | --- | --- | --- | --- | --- | --- | --- | --- | --- | --- |
| **vehicle** |  | P<0.001 | P<0.001 | P<0.001 | P<0.001 | P<0.001 | P<0.001 | P<0.001 | P<0.001 | P<0.001 |
| **1,25(OH)2D3** | P<0.001 |  | P<0.001 | P<0.001 | P<0.001 | P<0.001 | P<0.001 | P<0.001 | P<0.001 | P<0.001 |
| **20(OH)D3** | P<0.001 | P<0.001 |  | P<0.001 | P<0.001 | P<0.001 | P<0.001 | P<0.001 | P<0.001 | P<0.001 |
| **20,23(OH)2D3** | P<0.001 | P<0.001 | P<0.001 |  | P<0.001 | P<0.001 | P<0.001 | P<0.001 | P<0.001 | P<0.001 |
| **1,20(OH)2D3** | P<0.001 | P<0.001 | P<0.001 | P<0.001 |  | P<0.001 | P<0.001 | P<0.001 | P<0.001 | P<0.001 |
| **pD** | P<0.001 | P<0.001 | P<0.001 | P<0.001 | P<0.001 |  | P<0.001 | P<0.001 | P<0.001 | P<0.001 |
| **pL** | P<0.001 | P<0.001 | P<0.001 | P<0.001 | P<0.001 | P<0.001 |  | p>0.05 | P<0.001 | P<0.001 |
| **20(OH)pD** | P<0.001 | P<0.001 | P<0.001 | P<0.001 | P<0.001 | P<0.001 | p>0.05 |  | P<0.001 | P<0.001 |
| **7DHP** | P<0.001 | P<0.001 | P<0.001 | P<0.001 | P<0.001 | P<0.001 | P<0.001 | P<0.001 |  | p>0.05 |
| **20(OH)7DHP** | P<0.001 | P<0.001 | P<0.001 | P<0.001 | P<0.001 | P<0.001 | P<0.001 | P<0.001 | p>0.05 |  |

**D**. **Human HL-60 leukemia: number of NBT positive cells, 5 days**

|  | **vehicle** | **1,25**  **(OH)2D3** | **20(OH)**  **D3** | **20,23**  **(OH)2D3** | **1,20**  **(OH)2D3** | **pD** | **pL** | **20(OH)**  **pD** | **7DHP** | **20(OH)**  **7DHP** |
| --- | --- | --- | --- | --- | --- | --- | --- | --- | --- | --- |
| **vehicle** |  | P<0.001 | P<0.001 | P<0.001 | P<0.001 | P<0.001 | P<0.001 | P<0.001 | P<0.001 | P<0.001 |
| **1,25(OH)2D3** | P<0.001 |  | p>0.05 | p>0.05 | p>0.05 | p>0.05 | p>0.05 | P<0.01 | P<0.01 | P<0.05 |
| **20(OH)D3** | P<0.001 | p>0.05 |  | p>0.05 | p>0.05 | p>0.05 | p>0.05 | P<0.05 | P<0.05 | p>0.05 |
| **20,23(OH)2D3** | P<0.001 | p>0.05 | p>0.05 |  | p>0.05 | p>0.05 | p>0.05 | P<0.01 | P<0.1 | P<0.05 |
| **1,20(OH)2D3** | P<0.001 | p>0.05 | p>0.05 | p>0.05 |  | p>0.05 | p>0.05 | P<0.05 | P<0.01 | P<0.05 |
| **pD3** | P<0.001 | p>0.05 | p>0.05 | p>0.05 | p>0.05 |  | p>0.05 | p>0.05 | p>0.05 | p>0.05 |
| **pL3** | P<0.001 | p>0.05 | p>0.05 | p>0.05 | p>0.05 | p>0.05 |  | p>0.05 | P<0.05 | p>0.05 |
| **20(OH)pD3** | P<0.001 | P<0.01 | P<0.05 | P<0.01 | P<0.05 | p>0.05 | p>0.05 |  | p>0.05 | p>0.05 |
| **7DHP** | P<0.001 | P<0.01 | P<0.05 | P<0.01 | P<0.01 | p>0.05 | P<0.05 | p>0.05 |  | p>0.05 |
| **20(OH)7DHP** | P<0.001 | P<0.05 | p>0.05 | P<0.05 | P<0.05 | p>0.05 | p>0.05 | p>0.05 | p>0.05 |  |

**E**. **Human HL-60 leukemia: number of NBT positive cells, 7 days**

|  | **vehicle** | **1,25**  **(OH)2D3** | **20(OH)**  **D3** | **20,23**  **(OH)2D3** | **1,20**  **(OH)2D3** | **pD** | **pL** | **20(OH)**  **pD** | **7DHP** | **20(OH)**  **7DHP** |
| --- | --- | --- | --- | --- | --- | --- | --- | --- | --- | --- |
| **vehicle** |  | P<0.001 | P<0.001 | P<0.001 | P<0.001 | P<0.001 | P<0.001 | P<0.001 | P<0.001 | P<0.001 |
| **1,25(OH)2D3** | P<0.001 |  | P<0.01 | p>0.05 | P<0.05 | P<0.05 | P<0.001 | P<0.001 | P<0.001 | P<0.01 |
| **20(OH)D3** | P<0.001 | P<0.01 |  | p>0.05 | p>0.05 | p>0.05 | P<0.01 | P<0.001 | P<0.001 | p>0.05 |
| **20,23(OH)2D3** | P<0.001 | p>0.05 | p>0.05 |  | p>0.05 | p>0.05 | p>0.05 | P<0.05 | P<0.01 | p>0.05 |
| **1,20(OH)2D3** | P<0.001 | P<0.05 | p>0.05 | p>0.05 |  | p>0.05 | P<0.01 | P<0.001 | P<0.001 | p>0.05 |
| **pD3** | P<0.001 | P<0.05 | p>0.05 | p>0.05 | p>0.05 |  | p>0.05 | P<0.01 | P<0.01 | p>0.05 |
| **pL3** | P<0.001 | P<0.001 | P<0.01 | p>0.05 | P<0.01 | p>0.05 |  | P<0.01 | P<0.001 | p>0.05 |
| **20(OH)pD3** | P<0.001 | P<0.001 | P<0.001 | P<0.05 | P<0.001 | P<0.01 | P<0.01 |  | p>0.05 | P<0.01 |
| **7DHP** | P<0.001 | P<0.001 | P<0.001 | P<0.01 | P<0.001 | P<0.01 | P<0.001 | p>0.05 |  | P<0.01 |
| **20(OH)7DHP** | P<0.001 | P<0.01 | p>0.05 | p>0.05 | p>0.05 | p>0.05 | p>0.05 | P<0.01 | P<0.01 |  |

**F**. **Human HL-60 leukemia: absorbance, 5 days**

|  | **vehicle** | **1,25**  **(OH)2D3** | **20(OH)**  **D3** | **20,23**  **(OH)2D3** | **1,20**  **(OH)2D3** | **pD** | **pL** | **20(OH)**  **pD** | **7DHP** | **20(OH)**  **7DHP** |
| --- | --- | --- | --- | --- | --- | --- | --- | --- | --- | --- |
| **vehicle** |  | P<0.001 | P<0.001 | P<0.001 | P<0.001 | P<0.01 | p>0.05 | P<0.01 | p>0.05 | P<0.01 |
| **1,25(OH)2D3** | P<0.001 |  | p>0.05 | P<0.01 | p>0.05 | P<0.001 | P<0.001 | P<0.001 | P<0.001 | P<0.001 |
| **20(OH)D3** | P<0.001 | p>0.05 |  | P<0.01 | p>0.05 | P<0.001 | P<0.001 | P<0.001 | P<0.001 | p>0.001 |
| **20,23(OH)2D3** | P<0.001 | P<0.01 | P<0.01 |  | p>0.05 | P<0.05 | P<0.01 | P<0.01 | P<0.01 | p>0.01 |
| **1,20(OH)2D3** | P<0.001 | p>0.05 | p>0.05 | P>0.05 |  | p>0.05 | p>0.05 | p>0.05 | p>0.05 | p>0.05 |
| **pD3** | P<0.01 | P<0.001 | P<0.001 | P<0.05 | p>0.05 |  | P<0.05 | p>0.05 | P<0.01 | p>0.05 |
| **pL3** | p>0.05 | P<0.001 | P<0.001 | P<0.01 | p>0.05 | P<0.05 |  | P<0.05 | p>0.05 | P<0.05 |
| **20(OH)pD3** | P<0.01 | P<0.001 | P<0.001 | P<0.01 | p>0.05 | p>0.05 | P<0.05 |  | P<0.05 | p>0.05 |
| **7DHP** | p>0.05 | P<0.001 | P<0.001 | P<0.01 | p>0.05 | P<0.01 | p>0.05 | P<0.05 |  | P<0.05 |
| **20(OH)7DHP** | P<0.01 | P<0.001 | P<0.001 | P<0.01 | p>0.05 | p>0.05 | P<0.05 | p>0.05 | P<0.05 |  |

**G**. **Human HL-60 leukemia: absorbance, 7 days**

|  | **vehicle** | **1,25**  **(OH)2D3** | **20(OH)**  **D3** | **20,23**  **(OH)2D3** | **1,20**  **(OH)2D3** | **pD** | **pL** | **20(OH)**  **pD** | **7DHP** | **20(OH)**  **7DHP** |
| --- | --- | --- | --- | --- | --- | --- | --- | --- | --- | --- |
| **vehicle** |  | P<0.001 | P<0.01 | P<0.01 | P<0.01 | p>0.05 | P<0.01 | P<0.01 | P<0.01 | P<0.01 |
| **1,25(OH)2D3** | P<0.001 |  | P<0.01 | P<0.01 | P<0.01 | P<0.001 | P<0.01 | P<0.001 | P<0.01 | P<0.01 |
| **20(OH)D3** | P<0.01 | P<0.01 |  | p>0.05 | P<0.05 | P<0.01 | P<0.05 | P<0.05 | P<0.05 | p>0.05 |
| **20,23(OH)2D3** | P<0.01 | P<0.01 | p>0.05 |  | P<0.05 | P<0.01 | P<0.05 | P<0.05 | P<0.05 | p>0.05 |
| **1,20(OH)2D3** | P<0.01 | P<0.01 | P<0.05 | P<0.05 |  | P<0.05 | p>0.05 | P<0.01 | p>0.05 | P<0.01 |
| **pD3** | p>0.05 | P<0.001 | P<0.01 | P<0.01 | P<0.05 |  | P<0.01 | P<0.001 | P<0.05 |  |
| **pL3** | P<0.01 | P<0.01 | P<0.05 | P<0.05 | p>0.05 | P<0.01 |  | P<0.01 | p>0.05 | P<0.001 |
| **20(OH)pD3** | P<0.01 | P<00.01 | P<0.05 | P<0.05 | P<0.01 | P<0.001 | P<0.01 |  | P<0.01 | P<0.05 |
| **7DHP** | P<0.01 | P<0.01 | P<0.05 | P<0.05 | p>0.05 | P<0.05 | p>0.05 | P<0.01 |  | P<0.01 |
| **20(OH)7DHP** | P<0.01 | P<0.01 | p>0.05 | p>0.05 | P<0.01 |  | P<0.001 | P<0.05 | P<0.01 |  |

H. Human U937 leukemia: number of NBT positive cells, 5 days

|  | **vehicle** | **1,25**  **(OH)2D3** | **20(OH)**  **D3** | **20,23**  **(OH)2D3** | **1,20**  **(OH)2D3** | **pD** | **pL** | **20(OH)**  **pD** | **7DHP** | **20(OH)**  **7DHP** |
| --- | --- | --- | --- | --- | --- | --- | --- | --- | --- | --- |
| **vehicle** |  | P<0.01 | P<0.01 | P<0.001 | P<0.05 | P<0.01 | P<0.01 | P<0.01 | P<0.01 | P<0.01 |
| **1,25(OH)2D3** | P<0.01 |  | p>0.05 | p>0.05 | p>0.05 | p>0.05 | p>0.05 | p>0.05 | p>0.05 | p>0.05 |
| **20(OH)D3** | P<0.01 | p>0.05 |  | p>0.05 | p>0.05 | p>0.05 | p>0.05 | p>0.05 | p>0.05 | p>0.05 |
| **20,23(OH)2D3** | P<0.001 | p>0.05 | p>0.05 |  | P<0.05 | P<0.01 | P<0.05 | P<0.01 | P<0.05 | p>0.05 |
| **1,20(OH)2D3** | P<0.05 | p>0.05 | p>0.05 | P<0.05 |  | p>0.05 | p>0.05 | p>0.05 | p>0.05 | p>0.05 |
| **pD3** | P<0.01 | p>0.05 | p>0.05 | P<0.01 | p>0.05 |  | p>0.05 | p>0.05 | p>0.05 | p>0.05 |
| **pL3** | P<0.01 | p>0.05 | p>0.05 | P<0.05 | p>0.05 | p>0.05 |  | p>0.05 | p>0.05 | p>0.05 |
| **20(OH)pD3** | P<0.01 | p>0.05 | p>0.05 | P<0.01 | p>0.05 | p>0.05 | p>0.05 |  | p>0.05 | p>0.05 |
| **7DHP** | P<0.01 | p>0.05 | p>0.05 | P<0.05 | p>0.05 | p>0.05 | p>0.05 | p>0.05 |  | p>0.05 |
| **20(OH)7DHP** | P<0.01 | p>0.05 | p>0.05 | p>0.05 | p>0.05 | p>0.05 | p>0.05 | p>0.05 | p>0.05 |  |

I. Human U937 leukemia: number of NBT positive cells, 7 days

|  | **vehicle** | **1,25**  **(OH)2D3** | **20(OH)**  **D3** | **20,23**  **(OH)2D3** | **1,20**  **(OH)2D3** | **pD** | **pL** | **20(OH)**  **pD** | **7DHP** | **20(OH)**  **7DHP** |
| --- | --- | --- | --- | --- | --- | --- | --- | --- | --- | --- |
| **vehicle** |  | P<0.001 | P<0.01 | P<0.001 | P<0.01 | P<0.001 | P<0.01 | P<0.05 | P<0.001 | P<0.001 |
| **1,25(OH)2D3** | P<0.001 |  | P<0.01 | P<0.001 | P<0.01 | P<0.001 | P<0.001 | P<0.01 | P<0.001 | P<0.01 |
| **20(OH)D3** | P<0.01 | P<0.01 |  | p>0.05 | p>0.05 | p>0.05 | p>0.05 | p>0.05 | P<0.05 | p>0.05 |
| **20,23(OH)2D3** | P<0.001 | P<0.001 | p>0.05 |  | p>0.05 | P<0.01 | P<0.01 | p>0.05 | P<0.01 | p>0.05 |
| **1,20(OH)2D3** | P<0.01 | P<0.01 | p>0.05 | p>0.05 |  | p>0.05 | p>0.05 | p>0.05 | p>0.05 | p>0.05 |
| **pD3** | P<0.001 | P<0.001 | p>0.05 | P<0.01 | p>0.05 |  | p>0.05 | p>0.05 | p>0.05 | P<0.05 |
| **pL3** | P<0.01 | P<0.001 | p>0.05 | P<0.01 | p>0.05 | p>0.05 |  | p>0.05 | p>0.05 | P<0.05 |
| **20(OH)pD3** | P<0.05 | P<0.01 | p>0.05 | p>0.05 | p>0.05 | p>0.05 | p>0.05 |  | p>0.05 | p>0.05 |
| **7DHP** | P<0.001 | P<0.001 | P<0.05 | P<0.01 | p>0.05 | p>0.05 | p>0.05 | p>0.05 |  | P<0.05 |
| **20(OH)7DHP** | P<0.001 | P<0.01 | p>0.05 | p>0.05 | p>0.05 | P<0.05 | P<0.05 | p>0.05 | P<0.05 |  |

J. Human U937 leukemia: absorbance, 5 days

|  | **vehicle** | **1,25**  **(OH)2D3** | **20(OH)**  **D3** | **20,23**  **(OH)2D3** | **1,20**  **(OH)2D3** | **pD** | **pL** | **20(OH)**  **pD** | **7DHP** | **20(OH)**  **7DHP** |
| --- | --- | --- | --- | --- | --- | --- | --- | --- | --- | --- |
| **vehicle** |  | P<0.01 | P<0.01 | P<0.01 | P<0.05 | P<0.01 | p>0.05 | P<0.01 | p>0.05 | P<0.05 |
| **1,25(OH)2D3** | P<0.01 |  | p>0.05 | p>0.05 | p>0.05 | p>0.05 | P<0.05 | p>0.05 | P<0.05 | p>0.05 |
| **20(OH)D3** | P<0.01 | p>0.05 |  | p>0.05 | P<0.05 | p>0.05 | P<0.05 | p>0.05 | P<0.01 | P<0.05 |
| **20,23(OH)2D3** | P<0.01 | p>0.05 | p>0.05 |  | p>0.05 | p>0.05 | p>0.05 | p>0.05 | P<0.05 | p>0.05 |
| **1,20(OH)2D3** | P<0.05 | p>0.05 | P<0.05 | p>0.05 |  | p>0.05 | p>0.05 | p>0.05 | p>0.05 | p>0.05 |
| **pD3** | P<0.01 | p>0.05 | p>0.05 | p>0.05 | p>0.05 |  | p>0.05 | p>0.05 | P<0.05 | p>0.05 |
| **pL3** | p>0.05 | P<0.05 | P<0.05 | p>0.05 | p>0.05 | p>0.05 |  | P<0.05 | p>0.05 | p>0.05 |
| **20(OH)pD3** | P<0.01 | p>0.05 | p>0.05 | p>0.05 | p>0.05 | p>0.05 | P<0.05 |  | P<0.05 | p>0.05 |
| **7DHP** | p>0.05 | p>0.05 | P<0.01 | P<0.05 | p>0.05 | P<0.05 | p>0.05 | P<0.05 |  | p>0.05 |
| **20(OH)7DHP** | P<0.05 | P<0.05 | P<0.05 | p>0.05 | p>0.05 | p>0.05 | p>0.05 | p>0.05 | p>0.05 |  |

K. Human U937 leukemia: absorbance, 7 days

|  | **vehicle** | **1,25**  **(OH)2D3** | **20(OH)**  **D3** | **20,23**  **(OH)2D3** | **1,20**  **(OH)2D3** | **pD** | **pL** | **20(OH)**  **pD** | **7DHP** | **20(OH)**  **7DHP** |
| --- | --- | --- | --- | --- | --- | --- | --- | --- | --- | --- |
| **vehicle** |  | P<0.01 | P<0.01 | P<0.05 | P<0.05 | P<0.05 | P<0.01 | p>0.05 | p>0.05 | P<0.05 |
| **1,25(OH)2D3** | P<0.01 |  | p>0.05 | P<0.01 | P<0.01 | P<0.01 | p>0.05 | p>0.05 | p>0.05 | P<0.01 |
| **20(OH)D3** | P<0.01 | p>0.05 |  | P<0.01 | P<0.01 | P<0.01 | p>0.05 | P<0.01 | P<0.01 | P<0.01 |
| **20,23(OH)2D3** | P<0.05 | P<0.01 | P<0.01 |  | p>0.05 | P<0.05 | P<0.01 | P<0.05 | P<0.05 | p>0.05 |
| **1,20(OH)2D3** | P<0.05 | P<0.01 | P<0.01 | p>0.05 |  | p>0.05 | P<0.01 | p>0.05 | p>0.05 | p>0.05 |
| **pD3** | P<0.05 | P<0.01 | P<0.01 | P<0.05 | p>0.05 |  | P<0.01 | p>0.05 | P<0.01 | P<0.01 |
| **pL3** | P<0.01 | p>0.05 | p>0.05 | P<0.01 | P<0.01 | P<0.01 |  | P<0.01 | P<0.01 | P<0.01 |
| **20(OH)pD3** | p>0.05 | P<0.01 | P<0.01 | P<0.05 | p>0.05 | p>0.05 | P<0.01 |  | p>0.05 | p>0.05 |
| **7DHP** | p>0.05 | P<0.01 | P<0.01 | P<0.05 | p>0.05 | P<0.01 | P<0.01 | p>0.05 |  | p>0.05 |
| **20(OH)7DHP** | P<0.05 |  | P<0.01 | p>0.05 | p>0.05 | P<0.01 | P<0.01 | p>0.05 | p>0.05 |  |
