# Supplementary material for: Products of Vitamin D3 or 7-Dehydrocholesterol Metabolism by Cytochrome P450scc Show Anti-Leukemia Effects, Having Low or Absent Calcemic Activity
Source: PLoS One. 2010 Mar 26;5(3):e9907. doi: 10.1371/journal.pone.0009907 (PMC2845617; doi:10.1371/journal.pone.0009907)
Supplement: Table S3 — Docking scores of the biding of tested ligands to the VDR. (0.04 MB DOC) [file pone.0009907.s007.doc]

**Table S3**. Docking scores of the biding of tested ligands to the VDR

| Vitamin D analog | Docking score in VDR | Rank |
| --- | --- | --- |
| 1,25(H)_2_D3 (native ligand) | -13.5 | 1 |
| 1α, 20α(OH)_2_D3 | -12.9 | 2 |
| 1α, 20ß(OH)_2_D3 | -12.7 | 3 |
| 20ß, 23R(OH)_2_D3 | -12.1 | 4 |
| 20α, 23R(OH)_2_D3 | -11.9 | 5 |
| 20α(OH)D3 | -11.7 | 6 |
| 20ß(OH)D3 | -11.4 | 7 |
| pD | -11.2 | 8 |
| 20SpD | -11.0 | 9 |
| 20RpD | -11.0 | 10 |
