# Supplementary material for: Products of Vitamin D3 or 7-Dehydrocholesterol Metabolism by Cytochrome P450scc Show Anti-Leukemia Effects, Having Low or Absent Calcemic Activity
Source: PLoS One. 2010 Mar 26;5(3):e9907. doi: 10.1371/journal.pone.0009907 (PMC2845617; doi:10.1371/journal.pone.0009907)
Supplement: Table S4 — Primers used for real time RT-PCR analysis. (0.03 MB DOC) [file pone.0009907.s008.doc]

**Table S4.** Primers used for real time RT-PCR analysis.

| Gene | Primer sequence (left and right) |
| --- | --- |
| β ACTIN L 5`-CCAACCGCGAGAAGATGA-3`  R 5`-`CCAGAGGCGTACAGGGATAGC-3`  CD11b L 5`-AACCCCTGGTTCACCTCCT-3`  R 5`-CATGACATAAGGTCAAGGCTGT-3`  TRANSFERRIN L 5`-TTGAGAAAACAATGCAAAATGTG-3`  RECEPTOR R 5`-CCCAGTTGCTGTCCTGATATAGA-3` | |
